# 1-(3-Chloro­phen­yl)-3-(4-nitro­phen­yl)urea

**DOI:** 10.1107/S1600536810040201

**Published:** 2010-10-13

**Authors:** Ting Sun, Jing Li, Feng-Ling Yang

**Affiliations:** a College of Chemistry and Chemical Engineering, Xuchang University, Xuchang, Henan Province 461000, People’s Republic of China; b Institute of Surface Micro and Nano Materials, Xuchang University, Xuchang, Henan Province 461000, People’s Republic of China

## Abstract

In the title compound, C_13_H_10_ClN_3_O_3_, prepared by the reaction of 1-chloro-3-isocyanato­benzene with 4-nitro­benzenamine, the two substituent benzene rings are roughly coplanar [inter-ring dihedral angle = 8.70 (7)°]. In the crystal, mol­ecules make cyclic inter­molecular associations through two urea–nitro N—H⋯O hydrogen bonds, forming a chain structure [give chain direction] in which there are also weak inter­molecular C—H⋯Cl inter­actions. The urea O atom has only intra­molecular aromatic ring C—H⋯O associations.

## Related literature

For the bioactivity of urea derivatives, see: Wang *et al.* (2001 ▶); Song *et al.* (2008 ▶); Yip *et al.* (1986 ▶); Liu *et al.* (2005 ▶).
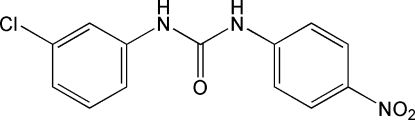


## Experimental

### 

#### Crystal data

C_13_H_10_ClN_3_O_3_
*M* *_r_* = 291.69
Monoclinic, 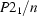

*a* = 8.3410 (13) Å
*b* = 12.5410 (18) Å
*c* = 12.1120 (16) Å
β = 99.866 (5)°
*V* = 1248.2 (3) Å^3^
*Z* = 4
Mo *K*α radiation
μ = 0.32 mm^−1^
*T* = 113 K
0.24 × 0.22 × 0.20 mm

#### Data collection

Rigaku Saturn724 CCD diffractometer
Absorption correction: multi-scan (*CrystalClear-SM Expert*; Rigaku, 2009 ▶) *T*_min_ = 0.928, *T*_max_ = 0.939
15672 measured reflections
2964 independent reflections
2396 reflections with *I* > 2σ(*I*)
*R*_int_ = 0.041

#### Refinement

*R*[*F* ^2^ > 2σ(*F* ^2^)] = 0.033
*wR*(*F* ^2^) = 0.092
*S* = 1.04
2964 reflections
189 parameters
H atoms treated by a mixture of independent and constrained refinement
Δρ_max_ = 0.41 e Å^−3^
Δρ_min_ = −0.24 e Å^−3^

### 

Data collection: *CrystalClear-SM Expert* (Rigaku, 2009 ▶); cell refinement: *CrystalClear-SM Expert*; data reduction: *CrystalClear-SM Expert*; program(s) used to solve structure: *SHELXS97* (Sheldrick, 2008 ▶); program(s) used to refine structure: *SHELXL97* (Sheldrick, 2008 ▶); molecular graphics: *CrystalStructure* (Rigaku, 2009 ▶); software used to prepare material for publication: *CrystalStructure*.

## Supplementary Material

Crystal structure: contains datablocks global, I. DOI: 10.1107/S1600536810040201/zs2070sup1.cif

Structure factors: contains datablocks I. DOI: 10.1107/S1600536810040201/zs2070Isup2.hkl

Additional supplementary materials:  crystallographic information; 3D view; checkCIF report

## Figures and Tables

**Table 1:** Hydrogen-bond geometry (Å, °)

*D*—H⋯*A*	*D*—H	H⋯*A*	*D*⋯*A*	*D*—H⋯*A*
N1—H1⋯O3^i^	0.807 (16)	2.211 (16)	3.0131 (14)	172.8 (16)
N2—H2⋯O2^i^	0.832 (14)	2.136 (14)	2.9448 (14)	164.1 (14)
C3—H3⋯O1	0.95	2.26	2.8720 (15)	121
C9—H9⋯O1	0.95	2.31	2.8833 (15)	118
C12—H12⋯Cl1^ii^	0.95	2.83	3.5465 (13)	133

Symmetry codes: (i) 
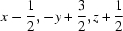
; (ii) 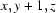.

**Fig. 1.**
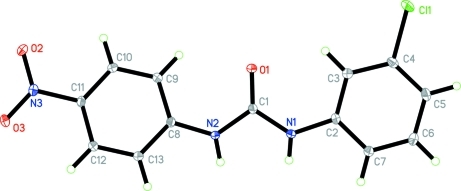
Molecular conformation and atom numbering scheme for the title compound, with displacement ellipsoids drawn at the 30% probability level.

**Fig. 2.**
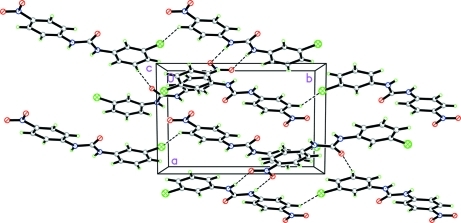
The packing diagram of the title compound. Intermolecular hydrogen bonds are shown as dashed lines.

## supplementary crystallographic information

### Comment

Previous studies have shown that urea derivatives have important medical and biological applications, e.g. *N, N*'-diarylurea derivatives have cytokinin activity (Wang *et al.*, 2001) and bacteriostatic activity. Compounds bearing a urea linkage to benzothiazole were also investigated for their ability to inhibit Raf-1 activity (Song *et al.*. 2008). Thidiazuron, a substituted heterocyclic urea compound, mimicked the effect of benzyladenine (BA) in the Ca^2+^ and cytokinin systems or on the IAA and cytokinin systems (Yip *et al.*. 1986). Recently, better activity was achieved with benzoyl urea derivatives (Liu *et al.*. 2005). In order to discover further biologically active urea compounds, the title compound C_13_H_10_ClN_3_O_3_ (I) was synthesized and its crystal structure is reported here.

In the structure of title compound (Fig. 1), the molecule is almost planar [torsion angles C1–N1–C2–C7 and C1–N2–C8–C13, 178.39 (11)° and -165.69 (11)°] with a dihedral angle between two phenyl rings of 8.70 (7)°. In the crystal structure, the molecules give cyclic intermolecular associations through two urea N–H···O_nitro_ hydrogen bonds (Table 1) giving a one-dimensional chain structure (Fig. 2) in which there are also weak intermolecular C—H···Cl interactions [C12–H12···Cl1^iii^, 3.5465 (13) Å] [symmetry code (iii): *x*, *y* + 1, *z*]. The urea O atom has only intramolecular aromatic ring C–H···O associations [C3–H3···O1, 2.8720 (15) Å; C9–H9···O1, 2.8833 (15) Å].

### Experimental

1-Chloro-3-isocyanatobenzene (0.153 g, 1 mmol) and 4-nitrobenzenamine (0.138 g, 1 mmol) were mixed and ground in an agate mortar, then irradiated by microwave for 1 min. After the reaction was completed, the resulting product was dissolved in 95% ethanol with warming and immediately filtered. The product obtained was recrystallized from ethanol and single crystals of the title compound were obtained by slow evaporation.

### Refinement

The urea H atoms were located by difference methods and their positional and isotropic displacement parameters were refined. Other H atoms were placed in calculated positions, with C—H = 0.95 Å, and included in the final cycles of refinement using a riding model, with *U*_iso_(H) = 1.2*U*_eq_(C).

### Crystal data

**Table:**

C_13_H_10_ClN_3_O_3_	*F*(000) = 600
*M**_r_* = 291.69	*D*_x_ = 1.552 Mg m^−^^3^
Monoclinic, *P*2_1_/*n*	Mo *K*α radiation, λ = 0.71075 Å
Hall symbol: -P 2yn	Cell parameters from 4351 reflections
*a* = 8.3410 (13) Å	θ = 1.6–27.9°
*b* = 12.5410 (18) Å	µ = 0.32 mm^−^^1^
*c* = 12.1120 (16) Å	*T* = 113 K
β = 99.866 (5)°	Prism, colorless
*V* = 1248.2 (3) Å^3^	0.24 × 0.22 × 0.20 mm
*Z* = 4	

### Data collection

**Table:**

Rigaku Saturn724 CCD diffractometer	2964 independent reflections
Radiation source: rotating anode	2396 reflections with *I* > 2σ(*I*)
multilayer	*R*_int_ = 0.041
Detector resolution: 14.222 pixels mm^-1^	θ_max_ = 27.9°, θ_min_ = 2.4°
ω scans	*h* = −10→10
Absorption correction: multi-scan (*CrystalClear-SM Expert*; Rigaku, 2009)	*k* = −16→16
*T*_min_ = 0.928, *T*_max_ = 0.939	*l* = −15→15
15672 measured reflections	

### Refinement

**Table:**

Refinement on *F*^2^	Primary atom site location: structure-invariant direct methods
Least-squares matrix: full	Secondary atom site location: difference Fourier map
*R*[*F*^2^ > 2σ(*F*^2^)] = 0.033	Hydrogen site location: inferred from neighbouring sites
*wR*(*F*^2^) = 0.092	H atoms treated by a mixture of independent and constrained refinement
*S* = 1.04	*w* = 1/[σ^2^(*F*_o_^2^) + (0.0589*P*)^2^] where *P* = (*F*_o_^2^ + 2*F*_c_^2^)/3
2964 reflections	(Δ/σ)_max_ = 0.001
189 parameters	Δρ_max_ = 0.41 e Å^−^^3^
0 restraints	Δρ_min_ = −0.24 e Å^−^^3^

### Special details

**Table:**

Geometry. All e.s.d.'s (except the e.s.d. in the dihedral angle between two l.s. planes) are estimated using the full covariance matrix. The cell e.s.d.'s are taken into account individually in the estimation of e.s.d.'s in distances, angles and torsion angles; correlations between e.s.d.'s in cell parameters are only used when they are defined by crystal symmetry. An approximate (isotropic) treatment of cell e.s.d.'s is used for estimating e.s.d.'s involving l.s. planes.
Refinement. Refinement of *F*^2^ against ALL reflections. The weighted *R*-factor *wR* and goodness of fit *S* are based on *F*^2^, conventional *R*-factors *R* are based on *F*, with *F* set to zero for negative *F*^2^. The threshold expression of *F*^2^ > σ(*F*^2^) is used only for calculating *R*-factors(gt) *etc*. and is not relevant to the choice of reflections for refinement. *R*-factors based on *F*^2^ are statistically about twice as large as those based on *F*, and *R*- factors based on ALL data will be even larger.

### Fractional atomic coordinates and isotropic or equivalent isotropic displacement parameters (Å^2^)

**Table:**

	*x*	*y*	*z*	*U*_iso_*/*U*_eq_	
Cl1	0.22721 (4)	0.01383 (3)	0.53821 (3)	0.02970 (12)	
O1	0.31522 (10)	0.39764 (6)	0.45111 (7)	0.0187 (2)	
O2	0.55192 (10)	0.80112 (7)	0.13369 (7)	0.0218 (2)	
O3	0.49009 (10)	0.93629 (7)	0.22967 (7)	0.0221 (2)	
N1	0.17484 (13)	0.41897 (8)	0.59747 (9)	0.0177 (2)	
N2	0.23742 (12)	0.56468 (8)	0.50077 (9)	0.0171 (2)	
N3	0.49071 (11)	0.83930 (8)	0.21073 (8)	0.0173 (2)	
C1	0.24839 (13)	0.45459 (9)	0.51100 (10)	0.0152 (2)	
C2	0.15298 (14)	0.31274 (9)	0.62949 (10)	0.0153 (2)	
C3	0.20047 (14)	0.22475 (9)	0.57217 (10)	0.0175 (3)	
H3	0.2525	0.2338	0.5089	0.021*	
C4	0.16951 (15)	0.12397 (9)	0.61013 (10)	0.0191 (3)	
C5	0.09396 (15)	0.10706 (10)	0.70225 (10)	0.0205 (3)	
H5	0.0743	0.0369	0.7262	0.025*	
C6	0.04799 (14)	0.19542 (10)	0.75831 (10)	0.0198 (3)	
H6	−0.0042	0.1858	0.8215	0.024*	
C7	0.07749 (14)	0.29736 (9)	0.72298 (10)	0.0175 (3)	
H7	0.0463	0.3573	0.7624	0.021*	
C8	0.29852 (14)	0.62892 (9)	0.42460 (10)	0.0151 (2)	
C9	0.34844 (14)	0.59016 (9)	0.32687 (10)	0.0177 (3)	
H9	0.3407	0.5162	0.3096	0.021*	
C10	0.40879 (14)	0.66018 (10)	0.25622 (10)	0.0178 (3)	
H10	0.4434	0.6348	0.1903	0.021*	
C11	0.41844 (14)	0.76771 (9)	0.28220 (10)	0.0157 (2)	
C12	0.36528 (14)	0.80862 (9)	0.37613 (10)	0.0174 (3)	
H12	0.3702	0.8830	0.3913	0.021*	
C13	0.30537 (14)	0.73906 (10)	0.44672 (10)	0.0174 (3)	
H13	0.2681	0.7657	0.5113	0.021*	
H1	0.1324 (18)	0.4614 (13)	0.6334 (14)	0.035 (5)*	
H2	0.1933 (16)	0.5948 (12)	0.5489 (12)	0.024 (4)*	

### Atomic displacement parameters (Å^2^)

**Table:**

	*U*^11^	*U*^22^	*U*^33^	*U*^12^	*U*^13^	*U*^23^
Cl1	0.0455 (2)	0.01399 (17)	0.0309 (2)	0.00282 (13)	0.01006 (16)	−0.00398 (12)
O1	0.0246 (5)	0.0139 (4)	0.0200 (4)	0.0023 (3)	0.0111 (4)	−0.0004 (3)
O2	0.0264 (5)	0.0220 (5)	0.0196 (4)	−0.0022 (4)	0.0115 (4)	0.0009 (4)
O3	0.0290 (5)	0.0128 (4)	0.0259 (5)	−0.0023 (3)	0.0089 (4)	0.0026 (3)
N1	0.0245 (6)	0.0118 (5)	0.0195 (5)	0.0016 (4)	0.0114 (4)	−0.0003 (4)
N2	0.0244 (6)	0.0115 (5)	0.0182 (5)	0.0011 (4)	0.0120 (4)	−0.0003 (4)
N3	0.0171 (5)	0.0172 (5)	0.0178 (5)	−0.0014 (4)	0.0035 (4)	0.0030 (4)
C1	0.0159 (6)	0.0137 (5)	0.0164 (5)	−0.0012 (4)	0.0037 (4)	0.0008 (4)
C2	0.0151 (6)	0.0132 (5)	0.0173 (6)	−0.0008 (4)	0.0019 (5)	0.0017 (4)
C3	0.0191 (6)	0.0168 (6)	0.0171 (6)	0.0007 (5)	0.0042 (5)	0.0000 (5)
C4	0.0220 (6)	0.0141 (6)	0.0202 (6)	0.0015 (5)	0.0011 (5)	−0.0023 (5)
C5	0.0235 (6)	0.0147 (6)	0.0228 (6)	−0.0032 (5)	0.0020 (5)	0.0037 (5)
C6	0.0197 (6)	0.0209 (6)	0.0192 (6)	−0.0023 (5)	0.0045 (5)	0.0043 (5)
C7	0.0187 (6)	0.0165 (6)	0.0179 (6)	0.0004 (5)	0.0050 (5)	0.0009 (4)
C8	0.0147 (6)	0.0142 (6)	0.0171 (5)	−0.0001 (4)	0.0046 (4)	0.0016 (4)
C9	0.0223 (6)	0.0137 (5)	0.0181 (6)	−0.0004 (5)	0.0067 (5)	−0.0010 (4)
C10	0.0208 (6)	0.0168 (6)	0.0172 (6)	0.0012 (5)	0.0072 (5)	−0.0006 (5)
C11	0.0160 (6)	0.0150 (6)	0.0168 (6)	−0.0009 (4)	0.0049 (5)	0.0034 (4)
C12	0.0205 (6)	0.0132 (5)	0.0194 (6)	−0.0001 (4)	0.0056 (5)	0.0000 (4)
C13	0.0212 (6)	0.0148 (6)	0.0176 (6)	0.0010 (5)	0.0076 (5)	−0.0010 (4)

### Geometric parameters (Å, °)

**Table:**

Cl1—C4	1.7441 (12)	C5—C6	1.3876 (17)
O1—C1	1.2187 (14)	C5—H5	0.9500
O2—N3	1.2345 (13)	C6—C7	1.3835 (16)
O3—N3	1.2380 (13)	C6—H6	0.9500
N1—C1	1.3759 (15)	C7—H7	0.9500
N1—C2	1.4080 (14)	C8—C13	1.4064 (16)
N1—H1	0.806 (16)	C8—C9	1.4070 (15)
N2—C8	1.3860 (15)	C9—C10	1.3800 (16)
N2—C1	1.3879 (15)	C9—H9	0.9500
N2—H2	0.832 (14)	C10—C11	1.3840 (17)
N3—C11	1.4483 (14)	C10—H10	0.9500
C2—C3	1.3968 (16)	C11—C12	1.3882 (16)
C2—C7	1.4001 (16)	C12—C13	1.3741 (16)
C3—C4	1.3842 (16)	C12—H12	0.9500
C3—H3	0.9500	C13—H13	0.9500
C4—C5	1.3885 (16)		
			
C1—N1—C2	127.77 (10)	C7—C6—C5	120.52 (11)
C1—N1—H1	119.4 (12)	C7—C6—H6	119.7
C2—N1—H1	112.7 (12)	C5—C6—H6	119.7
C8—N2—C1	127.69 (10)	C6—C7—C2	120.39 (11)
C8—N2—H2	117.4 (10)	C6—C7—H7	119.8
C1—N2—H2	114.8 (10)	C2—C7—H7	119.8
O2—N3—O3	122.45 (10)	N2—C8—C13	116.84 (10)
O2—N3—C11	118.67 (10)	N2—C8—C9	123.70 (11)
O3—N3—C11	118.88 (10)	C13—C8—C9	119.44 (11)
O1—C1—N1	124.88 (11)	C10—C9—C8	119.59 (11)
O1—C1—N2	124.04 (11)	C10—C9—H9	120.2
N1—C1—N2	111.08 (10)	C8—C9—H9	120.2
C3—C2—C7	119.89 (11)	C9—C10—C11	119.48 (11)
C3—C2—N1	123.33 (11)	C9—C10—H10	120.3
C7—C2—N1	116.78 (10)	C11—C10—H10	120.3
C4—C3—C2	118.12 (11)	C10—C11—C12	122.16 (11)
C4—C3—H3	120.9	C10—C11—N3	118.82 (10)
C2—C3—H3	120.9	C12—C11—N3	119.00 (11)
C3—C4—C5	122.85 (11)	C13—C12—C11	118.46 (11)
C3—C4—Cl1	118.29 (9)	C13—C12—H12	120.8
C5—C4—Cl1	118.85 (10)	C11—C12—H12	120.8
C6—C5—C4	118.22 (11)	C12—C13—C8	120.81 (11)
C6—C5—H5	120.9	C12—C13—H13	119.6
C4—C5—H5	120.9	C8—C13—H13	119.6
			
C2—N1—C1—O1	−4.21 (19)	C1—N2—C8—C13	−165.69 (11)
C2—N1—C1—N2	176.06 (11)	C1—N2—C8—C9	16.15 (18)
C8—N2—C1—O1	−0.55 (19)	N2—C8—C9—C10	−179.59 (11)
C8—N2—C1—N1	179.19 (11)	C13—C8—C9—C10	2.29 (17)
C1—N1—C2—C3	−2.48 (19)	C8—C9—C10—C11	−0.37 (17)
C1—N1—C2—C7	178.39 (11)	C9—C10—C11—C12	−1.71 (18)
C7—C2—C3—C4	0.46 (17)	C9—C10—C11—N3	176.47 (10)
N1—C2—C3—C4	−178.64 (11)	O2—N3—C11—C10	−5.16 (16)
C2—C3—C4—C5	−0.09 (18)	O3—N3—C11—C10	175.45 (10)
C2—C3—C4—Cl1	179.53 (9)	O2—N3—C11—C12	173.09 (10)
C3—C4—C5—C6	−0.06 (19)	O3—N3—C11—C12	−6.30 (16)
Cl1—C4—C5—C6	−179.68 (9)	C10—C11—C12—C13	1.80 (17)
C4—C5—C6—C7	−0.16 (18)	N3—C11—C12—C13	−176.39 (10)
C5—C6—C7—C2	0.54 (17)	C11—C12—C13—C8	0.20 (17)
C3—C2—C7—C6	−0.69 (17)	N2—C8—C13—C12	179.54 (10)
N1—C2—C7—C6	178.47 (11)	C9—C8—C13—C12	−2.22 (18)

### Hydrogen-bond geometry (Å, °)

**Table:**

*D*—H···*A*	*D*—H	H···*A*	*D*···*A*	*D*—H···*A*
N1—H1···O3^i^	0.807 (16)	2.211 (16)	3.0131 (14)	172.8 (16)
N2—H2···O2^i^	0.832 (14)	2.136 (14)	2.9448 (14)	164.1 (14)
C3—H3···O1	0.95	2.26	2.8720 (15)	121
C9—H9···O1	0.95	2.31	2.8833 (15)	118
C12—H12···Cl1^ii^	0.95	2.83	3.5465 (13)	133

Symmetry codes: (i) *x*−1/2, −*y*+3/2, *z*+1/2; (ii) *x*, *y*+1, *z*.
